# The Effect of Freezing Twice during Assisted Reproductive Technology on Perinatal and Neonatal Outcomes

**DOI:** 10.1155/2022/5623462

**Published:** 2022-04-04

**Authors:** Ye Pan, Richao Wu, Ze Wang, Xiufang Li, Shanshan Gao, Yuhua Shi

**Affiliations:** ^1^Center for Reproductive Medicine, Cheeloo College of Medicine, Shandong University, Jinan, Shandong 250012, China; ^2^Key Laboratory of Reproductive Endocrinology of Ministry of Education, Shandong University, Jinan, Shandong 250012, China; ^3^Shandong Key Laboratory of Reproductive Medicine, Jinan, Shandong 250012, China; ^4^Shandong Provincial Clinical Research Center for Reproductive Health, Jinan, Shandong 250012, China; ^5^National Research Center for Assisted Reproductive Technology and Reproductive Genetics, Shandong University, Jinan, Shandong 250012, China; ^6^Guangdong Provincial People's Hospital, Guangdong Academy of Medical Sciences, Guangdong, China

## Abstract

**Objective:**

The aim of this paper was to investigate whether two freeze-thaw cycles before embryo transfer may affect perinatal and neonatal outcomes.

**Materials and Methods:**

A total of 8,028 frozen-thawed embryo transfer patients who became pregnant between March 2013 and September 2019 were included. The patients were divided into two groups: the oocyte cryopreservation (OC) group (*N* = 96) and the control group (*N* = 7932). Propensity score matching (PSM) was used to adjust the baseline characteristics of the two groups at a proportion of 1 : 4. There were 96 patients in the OC group and 369 patients in the control group after PSM. The pregnancy-related complications and neonatal conditions after delivery of the two groups were compared.

**Results:**

The OC group had a higher stillbirth rate (3.1% vs. 0.3%, *P* = 0.029) than the control group after PSM. Moreover, a slightly higher pregnancy defect rate was found in the OC group. There was no significant difference in the rates of diabetes mellitus, hypertension during pregnancy, cesarean section, multiple births, low birth weight (LBW), or premature birth defects between the two groups.

**Conclusions:**

Our findings demonstrate that performing frozen-thawed embryo transfer (FET) with cryopreserved oocytes was associated with a higher rate of stillbirth than FET with fresh oocytes. The incidences of diabetes, gestational hypertension, cesarean section, multiple births, LBW, premature birth, and birth defects of the two groups were not significantly different.

## 1. Introduction

Cryopreservation technology has been widely used for embryo and gamete cryopreservation in assisted reproductive technology (ART), and the cryopreservation of oocytes and embryos is a major component of ART. The first successful birth from cryopreserved embryos was reported in 1983, and the first successful birth from cryopreserved oocytes was reported in 1986 [1, 2]. Early on, because oocytes were vulnerable to ice crystal damage and required more stringent freezing conditions than did embryos, the clinical application of oocyte cryopreservation (OC) was restricted [3]; however, the introduction and development of vitrification without any ice crystals has increased the possibilities regarding OC or embryo cryopreservation [4, 5].

Frozen-thawed embryo transfer (FET) can be used to store surplus embryos, increase the cumulative pregnancy rate, and reduce the risk of OHSS [6]. Many studies have shown that FET significantly improves the live birth rate and fertility quality compared to fresh embryo transfers [7, 8]. At present, FET is widely used in clinical applications, and the “freeze-all” strategy has been increasingly favored [9]. However, the safety of the embryo cryopreservation method remains controversial.

OC was originally used for females who needed to preserve their fertility due to medical conditions (cancer, premature ovarian failure, etc.), who could not obtain sperm on the day of oocyte retrieval, who needed to postpone childbirth, or who wished to become egg donors [3, 10]. It provides much more possibilities to increase cumulative pregnancy for patients to increase the chance of success of IVF. Additionally, with the rapid improvements in the educational levels and incomes of women globally, an increasing number of women, especially senior intellectuals, have become more willing to choose OC to delay fertility so that they have more time and more concentrated energy to engage in important work. OC for oocyte storage can prevent controversies caused by divorce or widowhood, reduce personal concerns or ethical issues, and increase women's reproductive autonomy [10].

To date, there have been few studies on pregnancy outcomes after OC. In recent years, research results have been inconsistent and controversial regarding whether cryopreserved oocytes have a lower success rate than fresh oocytes [11, 12]. However, in terms of the cumulative pregnancy rate, some scholars believe that OC could lead to a higher total cumulative pregnancy rate [13].

Patients are often concerned about whether FET or OC poses a risk to offspring perinatally or after birth. Regarding FET, there are consistent findings of an increased risk of hypertensive disorders of pregnancy and large for gestational age after FET, but the specific reasons for this are not clear [14–18]. Some studies have suggested that the risks might be relevant to FET, as FET may lead to epigenetic changes [19]. Regarding OC, some studies on the disease risk to offspring have OC confirmed that OC does not show an increased risk of adverse outcomes among offspring or of pregnancy complications; however, the research results and sample sizes were small, and the sufficiency of evidence needs to be further strengthened [20, 21].

For each OC patient, more than one oocyte is thawed in every cycle to maximize cost-effectiveness. Therefore, some patients with OC may have extra embryos to be frozen, while others with OC may need to have frozen embryos transferred for medical and personal reasons. Thus, these embryos would undergo two freeze-thawing cycles: freezing and thawing of the oocyte and freezing and thawing of the embryo. It is not clear whether two freeze-thaw cycles affects maternal or infant outcomes. To address this, we searched and screened the literature but were unable to retrieve relevant literature. Thus, we designed this study to explore this issue. The goals of our study were to determine whether twice freezing affects perinatal and neonatal outcomes and to help clinicians make informed decisions.

## 2. Materials and Methods

**Figure 1:**
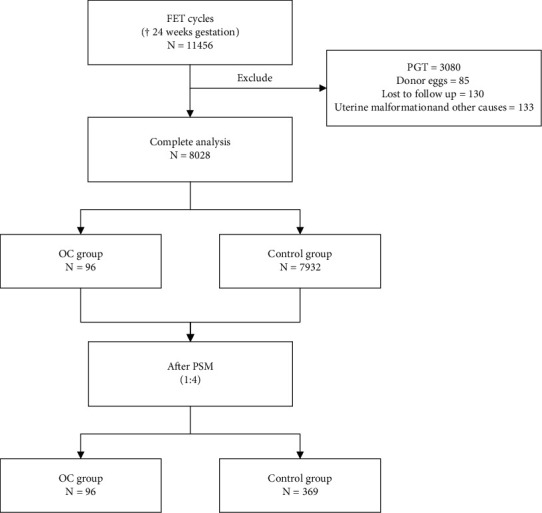
Flow chart for participants.

We selected all frozen-thawed embryo transferred patients at the Affiliated Reproductive Hospital of Shandong University who became pregnant from March 2013 to September 2019. All frozen-thawed oocytes were subjected to intracytoplasmic sperm injection (ICSI) and blastocyst transfer. To reduce potential confounding factors and improve the efficiency of propensity score matching (PSM), the inclusion criteria were as follows: (1) pregnancy ≥24 weeks; (2) underwent an ICSI cycle; and (3) transferred D5/D6 blastocysts. The exclusion criteria were as follows: (1) donated eggs; (2) preimplantation genetic testing; (3) uterine abnormalities; and (4) missing data. The patients were divided into two groups: the OC group and the control group. The patients in the OC group first underwent oocyte freezing and thawing and later underwent FET. The control group underwent FET only with fresh oocytes. After strict screening, we finally identified 8028 patients with frozen-thawed embryos transferred, including 96 patients in the OC group and 7932 patients in the control group. This study was carried out according to the Helsinki Declaration. Ethical approval was obtained from the ethical committee of the Center for Reproductive Medicine affiliated with Shandong University.  Figure 1 illustrates the patient selection process with a flowchart.

### 2.1. Study Protocol

Based on the condition of each patient, medical staff developed different ovulation protocols. Ovulation induction programs included a long gonadotropin releasing hormone (GnRH) agonist regimen, short GnRH agonist regimen, GnRH antagonist regimen, mild stimulation protocol, and natural regimen. Ovulation induction programs have been published in previous studies [22–26]. In this study, the ovarian response was evaluated based on the serum steroid hormone level and gynecological ultrasound results, and physicians adjusted the gonadotropin (Gn) dose according to the results. When two or more follicles exceeded 18 mm, 4000–10,000 IU human chorionic gonadotropin (HCG) is triggered. For patients assigned to the OC group, all oocytes were derived from the patients. Oocytes were frozen and thawed by means of the vitrification method described in previous reports [27]. Some or all oocytes obtained from each patient in the OC group were frozen. All oocytes from the two groups were fertilized by ICSI. The embryos were vitrified on the fifth or sixth day according to the development of the embryo.

Endometrial preparation was performed using a natural cycle regimen, hormone replacement cycle regimen, or ovarian stimulation cycle. Natural ovulation cycles were adopted for patients with regular ovulation, and hormone replacement or ovarian stimulation cycles were used for patients with anovulatory or irregular menstruation. Upon ovulation or when endometrial thickness exceeded 7 mm, the luteal phase support regimen was added according to the endometrial preparation protocol. The blastocysts were transferred on the fifth day after ovulation or progesterone injection. Luteal phase support lasted until 10 weeks of gestation. Follow-up of the patients was conducted regularly through telephone or outpatient visits during pregnancy and the perinatal period, with neonatal outcomes obtained.

### 2.2. Outcomes

The evaluation results included mainly pregnancy-related complications and the neonatal condition after delivery. Complications during pregnancy included stillbirths, multiple pregnancies, gestational diabetes mellitus, and gestational hypertension. Neonatal condition included gestational age, birth weight, and gender. Low birth weight was defined as birth weight < 2500 g, and macrosomia was defined as birth weight > 4500 g. All deliveries included live born and stillborn infants. Babies born included all live born infants. Birth defects were determined per the International Classification of Diseases (ICD) version 10 code.

### 2.3. Statistical Analyses

All data were statistically analyzed using SPSS 26.0 (IBM SPSS 26.0, SPSS Inc.). Categorical variables are expressed as percentages, and the chi square test and Fisher's exact test were used for analysis. As the data did not meet the assumption of a normal distribution, continuous variables are expressed as the median (25th percentile–75th percentile), and a nonparametric test was used. *P* < 0.05 indicated a significant difference. To reduce selection bias and eliminate the interference of confounding factors, the PSM method was used in this study, and the nearest neighbor PSM was employed to match the two groups. PSM was used mainly to match the baseline variables of the two groups, including age, body mass index (BMI), follicle stimulating hormone (FSH), luteinizing hormone (LH), number of oocytes retrieved, polycystic ovary syndrome (PCOS), endometrial preparation scheme for FET, natural cycle, program cycle, ovarian stimulation cycle, number of transferred embryos, endometrial thickness, endometrial thickness on HCG trigger day, and previous conception. The matching ratio for PSM was 1 : 4 within a caliper width of 0.1 for a satisfactory match.

## 3. Results

A total of 8,028 FET cycles undergoing ICSI, blastocyst transfer, and final deliveries between March 2013 and September 2019 were included in the analysis. There were 96 patients in the OC group and 7,932 patients in the control group. Before PSM, there were statistically significant differences in baseline characteristics between the two groups (Table 1). To address confounding factors between the two groups, we performed PSM analysis. There were 96 cases in the OC group and 369 cases in the control group after PSM. There were no statistically significant differences in the baseline characteristics of the two groups after PSM (Table 2).

The pregnancy outcomes of all deliveries (stillbirth and live birth) ≥24 weeks are shown in Table 3. The fetal death rate in the OC group was higher than that in the control group (3.1% vs. 0.3%, *P* = 0.029). The rates of gestational diabetes mellitus, hypertension during pregnancy, cesarean section, multiple births, birth weight, and gestational age were comparable between the two groups.

Of the all 100 deliveries in the OC group, 6 (6%) cases (live and stillbirth) were diagnosed with the following major birth defects at birth (Table 4): neurological malformation (1 case), facial malformation (1 case), one respiratory malformation (1 case), and musculoskeletal malformations (3 cases). Of the all 395 deliveries in the control group, 14 (3.5%) (live and stillbirth) were diagnosed with severe birth defects at birth (Table 4): cardiovascular malformation (6 cases), respiratory malformation (2 case), cleft lip and palate (1 case), gastrointestinal malformation (1 case), reproductive organ malformation (1 case), musculoskeletal malformation (2 case), and chromosome malformation (1 case). The incidence of birth defects in the OC group was higher, but the difference between the two groups was not significant (*P* > 0.05) (see Table 4).

## 4. Discussion

The results of this retrospective study showed that performing FET with cryopreserved oocytes was associated with a higher rate of stillbirth than performing FET with fresh oocytes. There was no significant difference between the two groups in the incidence of diabetes, gestational hypertension, cesarean section, multiple births, low birth weight (LBW), preterm birth, or birth defects.

The development of cryopreservation technology enables the long-term preservation of gametes. With the increasing popularity of cryopreservation of gametes, although the clinical effect is excellent, its safety is still controversial. Patients and doctors are most concerned about the health of mothers and newborns in ART. It has been suggested that frozen-thawed embryos or oocytes may be associated with increased genetic and epigenetic risks [28]. For ethical reasons, most studies are based on animals. Studies have shown that cryopreservation is related to extensive damage to the cell membrane, resulting in changes in the function and state of cells and mitochondria [29]. Animal studies have shown that cryopreservation may affect embryonic mitochondrial DNA mutations and DNA fragments [30, 31]. Studies on different species indicated that the gene expression of frozen thawed embryos was different from that of fresh embryos [32–35]. Human studies have found that cryopreservation of oocytes does not increase the incidence of embryonic aneuploidy [36, 37]. On the other hand, previous studies have emphasized that the freezing process may change the epigenetic state [28]. Cryopreservation may interfere with the epigenetics of embryos and oocytes, affect the expression of embryonic genes, and lead to changes in early placental and fetal development [19]. Studies have shown that the expression of imprinted genes and levels of DNA methylation change in cryopreserved fetuses and placentas in mice [38]. A genome-wide miRNA analysis of the human full-term placenta suggested that the expression of microRNA, an important epigenetic regulator of gene expression in FET, was different from that in fresh embryo transfer and natural pregnancy [39]. With the wide acceptance of OC, FET after OC has broad application prospects. At present, the risk of freezing to gametes is being widely studied by experts. However, whether there is an accumulative increase in risk to the fetus after two freeze-thaw is worthy of further investigation. Therefore, this study of whether freezing and thawing twice in OC affects patients affect perinatal and neonatal outcomes has substantial importance.

Many studies have demonstrated that FET has increased risks in terms of gestational age babies and hypertensive disorders of pregnancy [14–18, 40]. However, the specific mechanism of these pregnancy complications after FET remains unclear. Regarding the neonatal outcomes of FET, most studies have shown that there is no significant difference in the incidence of neonatal mortality or neonatal malformations between fresh and FET oocytes [3]. A meta-analysis conducted by Yang et al. showed that FET had no significant correlation with neonatal organ system malformations and that FET did not increase the risk of neonatal organ system malformations [17].

Clinical research data on cryopreserved oocytes are limited. However, the perinatal outcome data of cryopreserved oocytes are reassuring. Almost all the literature has reported that the vitrification of oocytes cannot increase the risk of adverse obstetric and perinatal outcomes [20, 21, 41, 42]. Relevant research conducted by Levi-Setti et al. suggested that in 2252 live births after OC, the incidence of stillbirth was 0.3%, and the incidence of congenital malformation was 0.9% [43]. Although OC has short-term and safe clinical outcomes, its long-term effect is unknown due to the lack of long-term follow-up data after birth. It is necessary to extend the follow-up time and call on more scholars to participate in relevant studies to further evaluate the long-term clinical efficacy of OC.

The results of this study showed that the stillbirth rate of frozen oocyte FET patients was higher than that of fresh oocyte FET patients. Early studies did not find any difference in perinatal mortality between the OC cycle and fresh oocyte cycle or between the fresh embryo cycle and FET cycle [40, 43]. We found that the rate of stillbirth with FET cycles in this study was similar to those of previous reports (0.3%-0.5%) [8, 44, 45]. The reason for the difference between the two groups is possibly due to two freeze-thaws or statistical selection bias of random findings. Therefore, this finding needs to be interpreted with caution, and large randomized controlled trials (RCT) are needed. In terms of neonatal malformations, the incidence of major malformations was higher in the OC group than in the control group, but the difference was not statistically significant.

There was no significant difference in the incidence of gestational diabetes mellitus, gestational hypertension, cesarean section, multiple births, LBW, or premature birth between the OC group and the control group. However, our results may be affected by the low numbers of related outcome events. Therefore, further large RCTs are needed to confirm the above hypothesis.

To the best of our knowledge, there is currently no similar study on the use of two freezing and thawing cycles. Our study also provides guidance for the clinical application of FET with cryopreserved oocytes. We suggest that caution be used in the selection of frozen-thawed embryos transplanted with cryopreserved oocytes. The advantage of this study is the use of PSM to control for confounding factors in a nonrandomized study and avoid confounding bias. However, there are a few limitations in this study. First, the number of patients in the OC group was relatively limited because the included patients comprised all deliveries and the inclusion criteria were strict. Second, due to the limited number of patients, we did not evaluate all pregnancy complications. Third, although PSM was used to minimize bias, we could not cover all relevant confounding factors. Therefore, there is still a risk of selection bias. More multicenter, large RCTs, and expanded sample sizes are needed to verify our results in the future.

## 5. Conclusion

In this study, we found that FET after cryopreservation of oocytes was significantly correlated with a higher stillbirth rate than FET with fresh oocytes. The incidences of diabetes, gestational hypertension, cesarean section, multiple births, LBW, premature birth, and birth defects between the two groups were not significantly different.

## Figures and Tables

**Table 1:** Baseline characteristics before propensity score matching.

Characteristic	Study group *N* = 96	Control group *N* = 7932	*P* value
Age (years)	29 (26-31)	29 (27-32)	0.075
BMI (kg/m^2^)	22.781 (19.995-25.038)	22.761 (20.569-25.390)	0.521
FSH (IU/L)	5.84 (4.98-7.038)	6.12 (5.272-7.14)	0.103
LH (IU/L)	5.175 (3.783-7.325)	5.04 (3.74-6.828)	0.558
Number of oocytes retrieved	19 (14-28)	15 (11-19)	0^a^
PCOS (%)	23 (23.96)	1434 (18.08)	0.137
Regimen of endometrial preparation for FET (%)			0^a^
Natural cycles	35 (36.46)	4441 (55.99)	
Programmed cycles	43 (44.79)	2756 (34.75)	
Ovarian stimulation cycles	18 (18.75)	735 (9.27)	
Number of transferred embryos (%)			0.209
1	85 (88.54)	6647 (83.80)	
2	11 (11.46)	1285 (16.20)	
Endometrial thickness on HCG trigger day (cm)	1 (0.9-1.1)	1 (0.85-1.1)	0.813
Previous conception (%)	23 (23.96)	2535 (31.96)	0.094

Values are the median (interquartile range) or number (percentage). ^a^Statistically significant. BMI: body mass index; FSH: follicle stimulating hormone; LH: luteinizing hormone; PCOS: polycystic ovary syndrome; FET: frozen embryo transfer; HCG: human chorionic gonadotropin.

**Table 2:** Baseline characteristics after propensity score matching.

Characteristic	Study group *N* = 96	Control group *N* = 369	*P* value
Age (years)	29 (26-31)	28 (26-31)	0.586
BMI (kg/m^2^)	22.781 (19.995-25.038)	22.609 (20.355-25.596)	0.773
FSH (IU/L)	5.84 (4.98-7.038)	5.96 (5.11-6.87)	0.694
LH (IU/L)	5.175 (3.783-7.325)	5.25 (3.965-6.975)	0.818
Number of oocytes retrieved	19 (14-28)	20 (15-25)	0.983
PCOS (%)	23 (23.96)	73 (19.8)	0.895
Regimen of endometrial preparation for FET			0.827
Natural cycles	35 (36.46)	146 (39.6)	
Programmed cycles	43 (44.79)	161 (43.6)	
Ovarian stimulation cycles	18 (18.75)	62 (16.8)	
Number of transferred embryos			0.983
1	85 (88.54)	327 (88.6)	
2	11 (11.46)	42 (11.4)	
Endometrial thickness on HCG trigger day (cm)	1 (0.9-1.1)	1 (0.85-1.1)	0.897
Previous conception	23 (23.96)	78 (21.1)	0.551

Values are the median (interquartile range) or number (percentage). ^a^Statistically significant. BMI: body mass index; FSH: follicle stimulating hormone; LH: luteinizing hormone; PCOS: polycystic ovary syndrome; FET: frozen embryo transfer; HCG: human chorionic gonadotropin.

**Table 3:** The pregnancy outcomes of two groups.

Variables	OC group *N* = 96	Control group *N* = 369	*P* value
Gestational diabetes mellitus, number/total number (%)	4 (4.2)	20 (5.4)	0.798
Hypertension during pregnancy, number/total number (%)	8 (8.3)	21 (5.7)	0.34
Cesarean section, cesarean, number/total number (%)	71 (74)	264 (71.5)	0.639
Stillbirth (%)	3 (3.1)	1 (0.3)	0.029^a^
Live birth (%)			0.334
Singleton livebirth	89 (92.7)	342 (92.7)	
Twin livebirth	4 (4.2)	26 (7.0)	
Babies born	*N* = 97	*N* = 394	
Gestational age (weeks)	39.286 (38.357-40.286)	39.143 (38-40)	0.173
Gestational age category, number/total number (%)			0.179
<37 weeks	7 (7.2)	52 (13.2)	
37-41 weeks	84 (86.6)	309 (78.4)	
≥41 weeks	6 (6.2)	33 (8.4)	
Birth weight (kg)	3.4 (3.1-3.7)	3.4 (3.0-3.7)	0.512
Macrosomia	5 (5.2)	36 (9.1)	0.204
LBW	13 (13.4)	54 (13.7)	0.938
Females	46 (47.4)	206 (52.3)	0.391

Values are the median (interquartile range) or number (percentage). ^a^Statistically significant. BMI: body mass index; FSH: follicle stimulating hormone; LH: luteinizing hormone; PCOS: polycystic ovary syndrome; FET: frozen embryo transfer; HCG: human chorionic gonadotropin.

**Table 4:** Congenital anomalies after FET with cryopreserved oocytes and fresh oocytes.

Variables	OC group	Control group
All deliveries^∗^	100	395
Major malformations	6 (6)	14 (3.5)
Congenital malformations of the nervous system (Q00-07)	1	0
Congenital malformations of eye, ear, face and neck (Q10-18)	1	0
Congenital malformations of the circulatory system (Q20-28)	0	6
Congenital malformations of the respiratory system (Q30-34)	1	2
Cleft lip and cleft palate (Q35-37)	0	1
Other congenital malformations of the digestive system (Q38-45)	0	1
Congenital malformations of genital organs (Q50-56)	0	1
Congenital malformations and deformations of the musculoskeletal system (Q65-79)	3	2
Chromosomal abnormalities, not elsewhere classified (Q90-99)	0	1

Values are numbers (percentages). ^∗^The number of all deliveries includes the number of live newborns plus stillborn babies.

## Data Availability

The dataset of this study is available from the corresponding author on request.
